# Alarmingly high prevalence of high-risk drug use among palestinian males: a cross-sectional study

**DOI:** 10.1186/s12888-023-05190-0

**Published:** 2023-09-26

**Authors:** Salwa Massad, Hadil Dalloul, Lina Adwan, Khalid Abu Saman, Rawan Kafri, Walaa Abu Alia, Marina Tucktuck, Lisa G. Johnston

**Affiliations:** 1 Palestinian National Institute of Public Health, Ramallah, Palestine; 2 Birzeit University, Birzeit, Palestine; 3 Primary Health Directorate, Ministry of Health, Ramallah, Palestine; 4 School of Medicine, St. George’s University, St. George’s, Grenada; 5 School of International Health and Tropical Medicine, LGJ Consultants, Tulane University, Louisiana, USA

**Keywords:** High-risk drug use, Respondent-driven sampling, Palestine, Tramadol, Synthetic marijuana

## Abstract

**Background:**

The unique socioeconomic context in Palestine, characterized by political and economic tensions, creates conditions that facilitate the spread of illicit drug use among Palestinians. This paper presents findings from a 2017 survey of high-risk drug use (HRDU) among males in four regions in Palestine: the West Bank (north, middle, and south) and the Gaza Strip. These findings are essential for developing effective policies to respond to the increasing use of drugs among Palestinians.

**Methods:**

Eligible participants were males aged 15 years and above who used at least one drug other than non-synthetic hashish or marijuana during the previous week. Participants underwent a face-to-face interview and had their drug use verified by urinalysis. Data were collected using respondent-driven sampling and data were analyzed using the successive sampling estimator. Multivariate regression analysis was conducted to examine factors associated with ever seeking rehabilitation services for illicit drug use in the West Bank and the Gaza Strip.

**Results:**

A total of 400 males who use drugs were sampled in Gaza, plus 299 in the south, 300 in the north, and 299 in the middle region of the West Bank. It is estimated that there are 26,500 male HRDUs in Palestine comprising 1.8% of the male population aged 15 and above. Findings indicate that polydrug use is a serious issue in Palestine, especially in the West Bank, and that synthetic marijuana is prevalent among teenagers and young adults.

**Conclusions:**

Palestine must strengthen its national efforts to scale up harm reduction and treatment and care options for people suffering from drug use disorders, especially those involved in polydrug use. Additional measures are needed to prevent substance use among children and youth, support the families of people who use drugs, and ensure the continuity of HRDU services during emergencies.

## Background

Drug use is criminalized, hidden, and stigmatized in most societies. Worldwide, an estimated 36.3 million people, or about 5.5% of the population aged between 15 and 64 years, used drugs at least once in 2020, and around 13% of all drug users suffered from drug use disorders, including dependence [1]. The Mediterranean region has been affected by the widespread use of illicit drugs [2]. Although the extent of illicit drug use in Arab countries is difficult to estimate due to the limited availability of research and the stigma associated with it [2]. Available data indicate abuse of Tramadol (the common brand name is Tramal, a drug that affects the central nervous system and used primarily to treat severe pain) in Egypt [3], heroin in Libya, cannabis and heroin in Morocco and Algeria, and different illicit drugs in Jordan [4]. In Saudi Arabia, the most commonly used illicit drugs are amphetamines, heroin, and cannabis, with trends increasing in the use of cannabis and amphetamines [5], Furthermore, the use of synthetic cannabinoids, also known as “Spice” and are typically sold as “herbal blends” or “incense”, are of growing concern. Spice products can have different potency based on number and types of additives which are known to increase the risk of unintentional overdose [6].

In Palestine, the unique socioeconomic context characterized by political and economic tensions has created conditions that facilitate the spread of illicit drug use. Some research indicates that Palestinian youth use illicit drugs to cope with the existing harsh conditions, which include political violence, house demolitions, arrests, restrictions on movement, and land encroachments [7]. The use of more than one drug simultaneously, or polydrug use, is a serious concern in Palestine. Factors associated with polydrug use include being younger, being younger at first drug use [4], and poor mental health [5]. Furthermore, despite the rising global incidence of drug addiction, there is still a treatment gap whereby only 10.6% of those in need of assistance received rehabilitation services [8]. Factors associated with seeking reahbilitation services for illicit drug use include support from family and friends, plus awareness of the availability of and accessibility to rehabilitative programs [8]. Currently, the West Bank has only three government drug treatment centers, including the Methadone Treatment Center, and five private centers, in addition to rehabilitation centers in Israel. In the Gaza Strip, there are four public rehabilitation centers and one newly established private rehabilitation center for women [9].

Drug use is exacerbated by the the absence of a unified Palestinian authority and police system, internal Palestinian conflict, weak enforcement of laws, and limited control of borders to combat the trafficking of illicit drugs. UN estimates from official sources indicate that there are about 10,000 registered drug users in the West Bank and the Gaza Strip, and about 15,000 in East Jerusalem [10]. Among heroin users, 40% are injecting [10]. The Anti Narcotic Department Annual Report 2016 found that drug seizures increased more than 2.5-fold between 2012 and 2016 (582 vs. 1437 respectively), along with the number of arrests for drug abuse violations (681 vs. 1754 respectively) in the West Bank [11].

It is essential to know the extent of HRDU and the pattern of illict drug consumption to allocate funding and develop effective rehabilitation services for people using drugs in Palestine. HRDU is defined as ‘recurrent drug use that causes actual harm (negative consequences) to the person (including dependence but also other health, psychological or social problems) or places the person at a high probability/risk of suffering such harm [12]. The aim of the study was to examine the extent and characteristics of HRDU in Palestine to inform the development of rehabilitation services in complex settings like Palestine. In addition, we used demographic data to determine the population size of men 15 years and above for use in gauging the size of the burden.

## Methods

Males aged 15 years and older who had used at least one drug other than non-synthetic hashish or marijuana during the week prior to the study, and living in either the north (Nablus), middle (Shufat camp) and south (Hebron) of the West Bank, or in Gaza City were sampled using respondent driven sampling in 2017. Hashish and marijuana were excluded from the study eligibility given the low morbidity and mortality associated with this drug and to better focus on higher risk drugs in order to inform the development of rehabilitation services in Palestine. Self-reported drug use in the previous week was verified by urinalysis. The study used the Abon Multi-Drug Test that screens for 10 types of drugs (Cocaine, Amphetamine, Methamphetamine, Cannabis, Methadone, Morphine, Phencyclidine, Barbiturates, Benzodiazepines, Tricyclic antidepressants) in addition to the Arco Biotech kit to screen for Tramadol. The West Bank, a large geographical area with long distances and checkpoints between major cities that indicate independent network components, had three distinct survey sites: Nablus in the north, Shufat camp in the middle region, and Hebron in the south. The survey in the Gaza Strip was conducted in Gaza City, the most populous city in Palestine.

Sample sizes were calculated using a change in proportion in drug use over time (0.032 for the West Bank, 0.128 for Jerusalem, and the 0.08 for the Gaza Strip, the mean of the proportion of the West Bank and Jerusalem), design effects of between 2 and 5, a power of 80%, and confidence of 95%. The resulting sample sizes were 300 in each of the three West Bank locations and 400 in Gaza City.

Given the stigma associated with drug use and findings from formative research that the population is socially networked, this survey used respondent-driven sampling (RDS) [13]. Briefly, RDS is a peer-to-peer recruitment method that optimizes a coupon quota and long recruitment chains to mimic a Markov Chain process [14, 15]. Recruitment began with four seeds (initial non-randomly selected participants) in Gaza City and Hebron, and three seeds in Shufat and Nablus. Seeds were selected via the Maqdesy Counselling Center in Jerusalem based on their ability to recruit peers who fulfilled a variety of different characteristics, including age, type of drug used, whether they injected drugs or not, and residential location. Seeds and subsequent participants were screened for eligibility, given a description of the survey process, underwent informed consent and had their urine tested with Abon by Alere (USA) and the Arco Biotech kit to confirm at least one of the following drugs in their system: cocaine, amphetamines, methamphetamines, methadone, morphine, phencyclidine, barbiturates, benzodiazepines, tricyclic anti-depressants or Tramadol. Once enrolled, participants completed face-to-face interviews by trained interviewers in Arabic. The study questionnaire was based on the WHO RDS tool [13]. As part of the survey questionnaire, participants were asked their personal network size in the following cascade of questions: How many people do you know, and who know you, who use drugs other than weed/marijuana and live in the study area? Of those, how many are 15 years of age or older? Of those, how many have you met in the last two weeks? Responses to these questions are necessary to weight the data during data analysis.

Once the survey steps were complete, each participant received up to three coupons with unique identification numbers to use in recruiting eligible peers. Successive waves of recruitment continued until the sample sizes were achieved [9].

This study also used successive sampling population size estimation (SSPSE), a Bayesian technique to estimate the sizes of hidden populations using data collected in a RDS survey. This method harnesses information about the network size with prior knowledge about population size and assumptions related to successive sampling to generate a median and mean estimation with probability bounds [16]. Participation was anonymous and no personal identifying information was collected. The survey was reviewed and approved by the Palestinian Ministry of Health Ethics Review Committee in 2016. All methods were carried out in accordance with relevant guidelines and regulations. For this study, parents’ consent was not taken for those below the age of 16 because of their higher risk and the fact that they are practicing stigmatizing behaviors and that many are considered ‘emancipated minors’.

### Data analysis

Data for each of the four locations were weighted and analyzed using the Giles successive sampling estimator (20) in RDS Analyst (www.hpmrg.org). Aggregate analysis was conducted on all four locations using SPSS along with exported successive sampling weights and population size weights to examine the prevalence of illicit drug use in combination with alcohol in the West Bank, and factors associated with seeking rehabilitation services. Bivariate and multivariable regression analysis were conducted to examine factors associated with ever seeking rehabilitation services for illicit drug use in the West Bank and in the Gaza Strip. Polydrug use was measured as the combined use of two or more of the following drugs based on traces found in the urinalysis: cocaine, amphetamines, methamphetamines, cannabis, methadone, morphine, phencyclidines, barbiturates, benzodiazepines, tricyclic anti-depressants, and/or Tramadol, or a drug combination with alcohol problem use [17]. Alcohol problem use was assumed for persons who reported having three or more alcoholic drinks per day [18]. HRDU was calculated based on polydrug use and/or methadone (MTD) or morphine (MOP) and/or Tramadol. Drug combinations with alcohol were calculated for participants who were considered to have an alcohol problem use in addition to two different drug combinations. Population size estimates using SS-PSE were calculated in RDS Analyst using the date of enrollment, personal network size, visibility imputation, and prior size data [16].

## Results

### Study sample

**Fig. 1:**
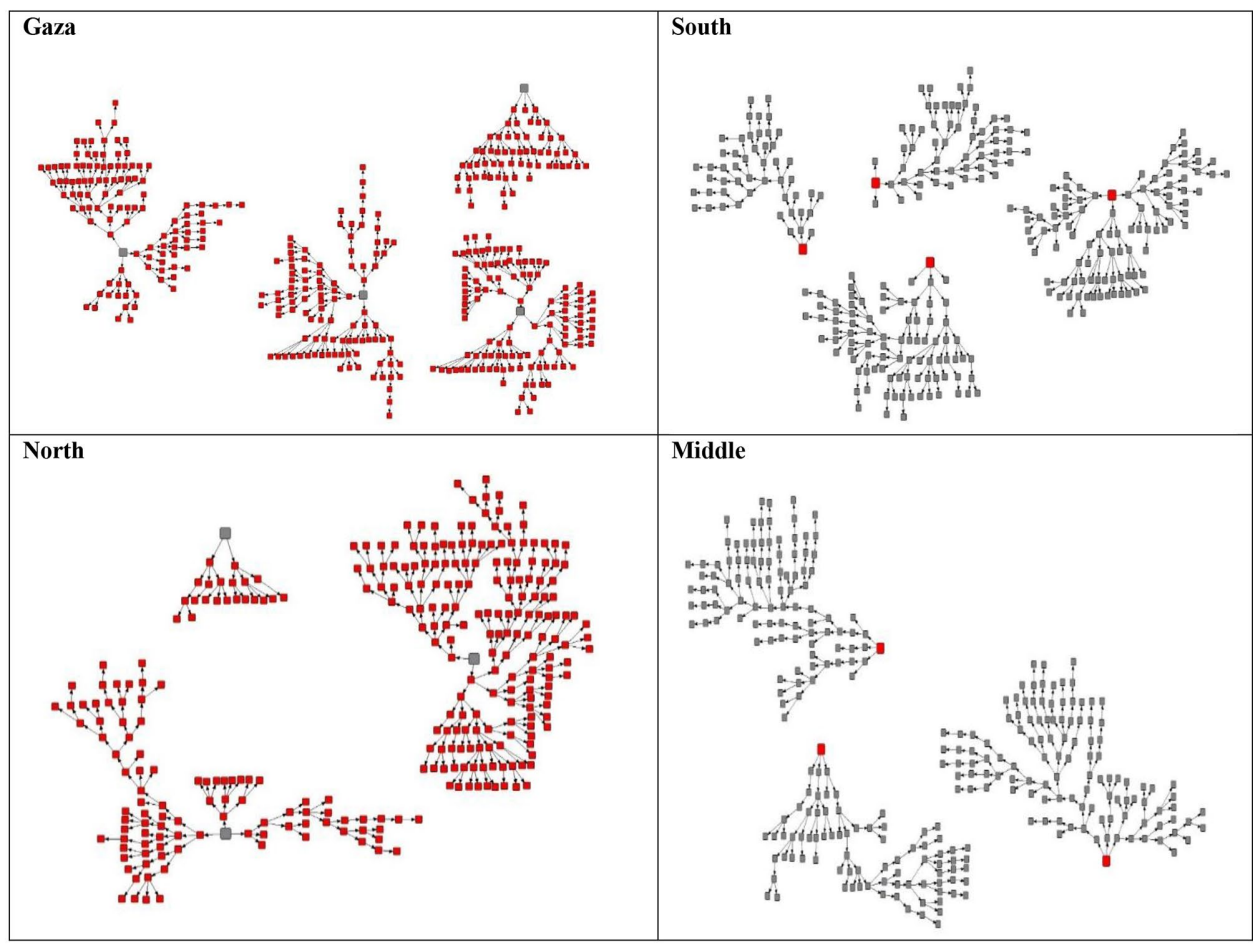
Recruitment graphs of HRDUs by study site, 2017

The final sample sizes were 400 in Gaza, 300 in the north of the West Bank, and 299 in each of the south and middle locations of the West Bank. The maximum number of waves was nine in Gaza, ten in the south, 13 in the north, and 14 in the middle of the West Bank (Fig. 1).

Almost all illicit drug users (95.5%) are HRDUs (99% in the Gaza Strip, 96% in the south, 93.3% in the north, and 97.3% in the middle). The median age of HRDUs in Palestine was between 22 and 29 years (Table 1). Almost all had attended school with the majority having only attended primary and secondary school. While the majority in the West Bank were single and never married, the majority in the Gaza Strip were married. Average monthly income ranged from 322 USD in the north to 871 USD in the middle region of the West Bank.

### Drug use

The first drugs used were synthetic marijuana in the West Bank and Tramadol in the Gaza Strip. Most HRDUs reported using Tramadol (99%) and Lyrica (active ingredient is pregabalin), an anti-convulsant drug also used for diabetic neuropathy (54%), in the Gaza Strip, and synthetic marijuana in the West Bank in the previous month.

**Fig. 2:**
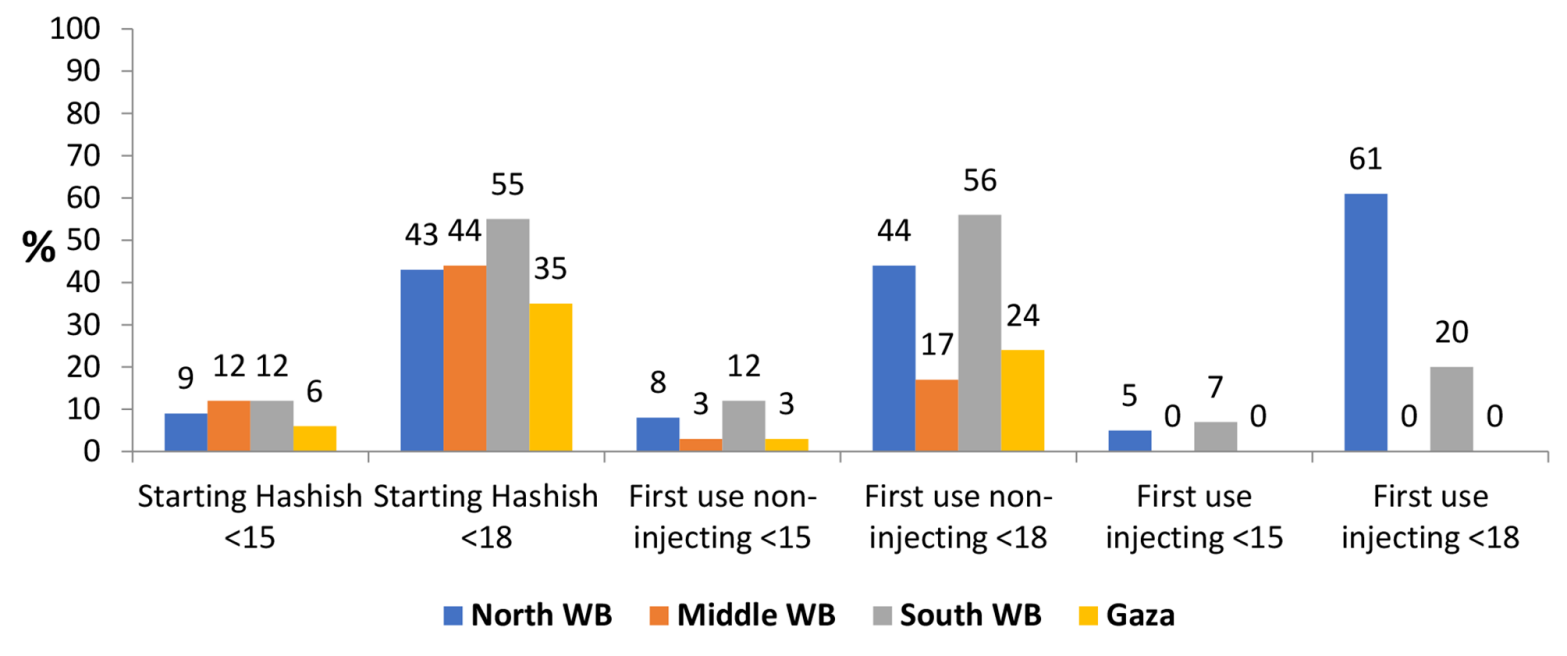
First use of drugs below 15 and below 18, by study site, 2017

Drug use during the previous week was more frequent in Gaza and the middle region compared with the south and north. Most HRDUs in Gaza, the south and middle were alone the last time they had used drugs. 44% of HRDU in the north, 17% in the middle, 56% in the south, and 24% in Gaza reported their first non-injecting drug use (other than hashish/marijuana) when they were younger than 18 years (Fig. 2).

**Table 1:** Demographics and drug use among HRDUs, Palestine, 2017

	GAZA STRIP		WEST BANK							
	**Gaza N = 400**		**South N = 299**		**North N = 300**			**Middle N = 299**		
	** N**	**%, (95% CI)**	**N**	**%, (95% CI)**	**N**	**%, (95% CI)**		**N**	**%, (95% CI)**	
Age (median) 26				24		**22**			**29**	
Attended school										
	394	98.6 (97.6,99.6)	288	96.3 (93.7,98.8)	295	98.4 (96.1,100)		291	97.6 (95.9,99.2)	
Education years: median										
	394	10	289	9	295	11		291	10	
Current marital status										
Single, engaged, permanent relationship	104	25·8 (20.9,30.7)	84	29·3 (23.9,34.7)	161	52·0 (45.2,58.9)		73	24.5 (18.9,30·0)	
Single, temporary relationship	60	15·0 (11.4,18.6)	102	34.4 (28·7,40.0)	54	17·9 (12.4,23.5)		74	24·5 (19.5,29.6)	
Married	222	55.7 (49.8,61.4)	108	34 (28.0,40.0)	77	28·0 (21.5,34.4)		130	43.8 (37.7,50.0)	
Divorced, separated, widowed	14	3.5 (1.7,5.1)	5	2·3 (0,4.8)	8	2·1 (0.5,3.6)		22	7·2 (4.3,10.1)	
Average monthly income (USD); Median										
	397	258	276	645	297	322		297	871	
Drugs detected by urinalysis										
Tramadol	386	96.1 (92.7,99.4)	13	3.9 (1.6,6.3)	20	7.2 (3.2,11.3)		136	40.1 (31.3,48.9)	
Marijuana	124	28.2 (22.2,34.3)	220	75.4 (70.4,80.3)	30	8.7 (5.2, 12.2)		102	30.5 (22.9,38.0)	
Benzodiazepines	88	22.3 (16.8,27.8)	193	64.1 (58.1,70.2)	209	73.7 (68.4,79.0)		117	29.4 (19.7,39.1)	
Amphetamines	11	1.1 (0.6,1.6)	8	2.0 (0.6,3.4)	185	67.2 (60.6,73.8)		145	53.6 (42.0,65.1)	
Methamphetamine	30	12.5 (6.4,18.5)	46	13.1 (9.3,16.9)	63	21.5 (16.2,26.9)		130	44.5 (35.3,53.3)	
Tricyclic anti-depressants	51	18.1 (12.4,23.7)	55	19.7 (14.8,24.6)	14	3.7 (1.6,5.8)		80	21.6 (14.3,28.7)	
Phencyclidine	46	10.6 (6.5,14.8)	38	12.2 (8.4,16.1)	25	7.3 (4.3,10.2)		19	5.3 (1.6,9.0)	
Barbiturates	5	2.8 (-0.7,06.2)	24	8.9 (5.1,12.6)	27	9.2 (5.5,13.0)		25	6.4 (2.8,9.9)	
Morphine	1	0.1 (0,0.37)	41	12.1 (8.2,16.0)	28	7.8 (4.8,10.6)		36	12.5 (6.7,18.1)	
Methadone	2	0.6 (0,1.6)	26	9.4 (5.3,13.5)	27	8.2 (4.7,11.7)		59	15.6 (9.6,21.5)	
Cocaine	0	-	3	0.7 (0.05,1.3)	19	4.3 (2.2,6.5)		195	58.8 (49.0,68.4)	
First type of non-injection drug used										
Tramadol	371	93.6 (91.2,96.0)	**1**	0.4 (0,1.2)	**2**	1.3 (0,2.6)	**1**			0.2 (0,0.6)
Weed	0	--	**185**	60.2 (53.3,67.1)	**104**	33.2 (26.9,39.4)	**45**			15.6 (10.8,20.5)
Synthetic Marijuana	0	--	**83**	28.6 (21.8,35.4)	**170**	58.4 (51.9,65.0)	**130**			42.0 (35.6,48.6)
Hallucinogenic	5	1.2 (0.2,2.2)	**3**	1.2 (0,2.9)	**0**	--	**37**			13.9 (9.5,18.3)
Heroin	6	1.3 (0.2,2.6)	**9**	3.6 (1.2,6.0)	**3**	0.7 (0,1.6)	**35**			11.8 (7.7,15.7)
Anxiolytics^1^	1	0.3 (0,0.8)	**0**	--	**0**	--	**16**			5·3 (2.7,8.0)
Steroids	0	--	**4**	0.3 (0,0.6)	**0**	--	**0**			
Crack	7	0.2 (0,0.8)	**1**	0.3 (0,0.8)	**0**	--	**14**			3.9 (2.0,5.9)
Cocaine	0	--	**6**	2.2 (0.4,3.9)	**0**	--	**1**			0.4 (0,1.2)
Sedatives	1	0.5 (0,1.2)	**1**	0.4 (0,1.2)	**5**	1.3 (0.2,2.4)	**4**			1.5 (0,3.8)
Crystal^2^	1	0.2 (0-0.5)	**5**	1.7 (0·1,3.3)	**1**	0.4 (0,1·0)	**0**			--
Phencyclidine^3^	0	--	**0**	--	**7**	2.5 (0.7,4.3)	**0**			--

### Drug use in the week prior the study survey

Based on urinalysis results, the most used drug was Tramadol (96%) in Gaza, benzodiazepines (74%) and amphetamines (67%) in the north most, marijuana (75%) and benzodiazepines (64%) in the south and cocaine (59%) and amphetamines (54%) in the middle region (Table 1).

Although urinalysis does not test for all possible drugs and some drugs cannot be detected in the urine after 48 h of intake, we found that over 90% HRDUs in all areas used two or more drugs, with significant variability per study location (p = 0·046 based on chi square test); polydrug use was 38.9% in the Gaza Strip. Based on urinalysis, HRDU in the north had a maximum number of 12 drugs and HRDUs in the middle and south had a maximum of eight drugs detected in their urine. The maximum number of drugs detected in the Gaza Strip was six. In addition, the most common drug combinations in the West Bank were cannabis and benzodiazepines (213); amphetamines and benzodiazepines (166); cocaine and amphetamines (121); while drug combinations with alcohol included amphetamines and benzodiazepines (103); and cannabis and benzodiazepines (88). In the Gaza Strip, the most common drug combinations were cannabis and Tramadol (122); benzodiazepines and Tramadol (80); and tricyclic anti-depressants and Tramadol (49) (Table 2). Although the urinalysis did not detect the use of synthetic marijuana, its use in the previous month was reported by 65% of HRDUs in the middle of the West Bank, 83% in the north and 89% in the south.

**Table 2:** Drug combinations with alcohol, West Bank, N = 898, 2017

Drug combinations with alcohol	Number	% (95% CI)
Amphetamines + Benzodiazepines	103	11.5 (9.5,13.8)
Cannabis + Benzodiazepines	88	9.8 (8.0,12.0)
Methamphetamines + Benzodiazepines	64	7.1 (5.6,9.1)
Amphetamines + Methamphetamines	59	6.6 (5.1,8.4)
Benzodiazepines + Tramadol	43	4.8 (3.5,6.5)
Morphine + Benzodiazepines	42	4.7 (3.4,6.3)
Benzodiazepines + Tricyclic Anti-depressants	39	4.3 (3.1,6.0)
Cocaine + Amphetamines	37	4.1 (3.0,5.7)
Methamphetamines + Cannabis	37	4.1 (3.0,5.7)
Cocaine + Benzodiazepines	33	3.7 (2.6,5.2)
Amphetamines + Cannabis	33	3.7 (2.6,5.2)
Cannabis + Tricyclic Anti-depressants	33	3.7 (2.6,5.2)

### Injecting drug use

Few HRDUs ever injected drugs. Among the 8% in the West Bank and 2% in Gaza who ever injected drugs, the majority in Gaza injected cocaine, whereas the majority in the south and middle injected heroin. In the north, almost equal percentages injected heroin and cocaine.

### Ever seeking treatment for drug use

Between 6% in the north and 27% in Gaza ever sought treatment for drug use. The median frequency of trying to stop using non-injection drugs in each of the four areas ranged from two to three times with a maximum range of 17 in the north and 50 in the middle region (not in table). 40% of HRDUs in Gaza, one third in the north and middle, and 17% in the south had ever been arrested for drug use.

Of those who reported ever injecting drugs, few in any location reported knowing of organizations and/or programs in their home city that assist people who inject drugs or offer information on HIV. Among those who inject drugs, a higher percentage in the middle region (44%, 11/28) reported ever receiving substance use treatment in a medical center for overdosing compared to those in the north (11%, 2/21), south (23%, 7/25), and Gaza (10%, 1/10) (Table 3).

Most HRDUs reported receiving no rehabilitation treatment services while in prison. Of the HRDUs who did report receiving rehabilitation treatment while in prison or detention, the majority received only medical rehabilitation treatment. Most HRDUs who had ever been arrested for drug use suffered withdrawal symptoms while in prison or detention.

**Table 3:** Rehabilitation treatment for illicit drug use by location, 2017

	GAZA STRIP		WEST BANK					
	**Gaza N = 400**		**South N = 299**		**North N = 300**		**Middle N = 299**	
	** N**	**%, (95% CI)**	**N**	**%, (95% CI)**	**N**	**%, (95% CI)**	**N**	**%, (95% CI)**
Ever sought rehabilitation treatment services for non-injecting drug use								
	106	27.3 (22.7,31.7)	36	11.6 (7.3,15.9)	20	5.6 (3.2,8.0)	74	24.5 (19.1,29.9)
Ever sought rehabilitation treatment services for injection drug use								
	**Gaza N = 7**		**South N = 25**		**North N = 21**		**Middle N = 28**	
-Treated at medical center for an overdose	2	21.9 (5.3, 38.8)	9	28.3 (0,68.3)	2	7.0 (0,18.5)	13	46.2 (25.8,66.6)
-Received rehabilitation treatment while in prison	1	10.2 (3.3, 17.1)	7	23.2 (6.7,39.7)	2	11.3 (6.0,16.7)	11	43.8 (30.5,56.8)
-Medical rehabilitation treatment	0	--	15	83.2 (69.6,96.7)	13	76.5^^	12	83.7 (58.5,100)
-Psychosocial rehabilitation treatment	0	--	4	16.8 (3.2,30.4)	6	23.5^^	3	16.3 (0,41.5)
	**Gaza N = 107**		**South N = 55**		**North N = 64**		**Middle N = 99**	
Experienced withdrawal symptoms while in prison for drug use	92	88.4 (79.7,0.97)	46	84.8 (73.7,95.9)	54	88.9 (83.7,93.9)	64	65.6 (54.1,77.1)

Based on univariate analysis (Table 4), factors associated with seeking rehabilitation services in the West Bank were older age, being married, having children, polydrug use, having been imprisoned, suffering from health conditions, memory loss, and withdrawal symptoms due to drug use. In the Gaza Strip, factors associated with seeking rehabilitation services were older age, having been imprisoned, having children, and suffering from withdrawal symptoms due to drug use. Based on multivariate logistic regression (Table 4), adjusting for confounders (being married and have children), factors associated with seeking rehabilitation services in the West Bank were older age, having been imprisoned, and suffering from memory loss due to drug use. In the Gaza Strip, factors associated with seeking rehabilitation services were having been imprisoned and suffering from withdrawal symptoms due to drug use.

**Table 4:** Factors associated with seeking rehabilitation services in the West Bank and the Gaza Strip, 2017

	Gaza Strip		West Bank	
	**Bivariate**	**Multivariate**	**Bivariate**	**Multivariate**
	OR (95% CI)	Adjusted OR (95% CI)	OR (95% CI)	Adjusted OR ¥ (95% CI)
Socio-demographic characteristics				
Age	1.0 (1.0, 1.1)*		1.1(1.0, 1.1)*	1.1 (1.0,1.1)*
Married	1.4 (0.9, 2.2)		3.5 (2.4, 5.1)*	
Have children	1.7 (1.0, 2.6)*		2.8 (1.1,7.4)*	
Suffering from consequences of drug use				
Been imprisoned	2.2 (1.3, 3.7)*	2.1 (1.2, 3.5)*	2.6 (1.7,4.0)*	2.31 (1.5, 3.6)*
Suffering from memory loss	1.1 (0.7,1.7)		2.9 (1.7,4.4)*	2.21 (1.4, 3.5)*
Suffering from withdrawal symptoms	7.1 (2.8,18.2)*	6.9 (2.7, 17.5)*	1.6 (1.1,2.5)*	

¥: Adjusted for being married and have children.

*: p-value < 0.05.

### Population size estimations

In both the West Bank and the Gaza Strip, HRDUs were found to comprise 1.8% of the male population aged 15 and above (total number: 16,453 in the West Bank, 10,047 in the Gaza Strip). From this information, the estimated number of HRDUs who ever injected drugs is 241 in the Gaza Strip, plus 532 in the north, 464 in the south, and 354 in the middle of the West Bank.

## Discussion

This study found that HRDU is prevalent throughout Palestine. We estimated that there are approximately 26,500 HRDUs in Palestine, comprising 1.8% of the male population aged 15 and above. The 2017 United Nations Office on Drugs and Crime (UNODC) Global Drug Report claims that the number of illicit drug users (i.e., used any kind of illicit drugs at least once in the past year) is 7–10 times higher than the number of HRDUs. Adding 7% on top of our size estimation of 1.8% (26,500 HRDUs) would indicate that there are at least 9.0% (180,000) males who use illicit drugs aged 15 years and older in Palestine. It is worth noting that the estimated prevalence of ever drug use is much higher than the global prevalence of 5% of people (males and females) between the ages of 15 and 64 years who used drugs at least once in 2015, based on the UNODC Global Report 2017 [1].

Political and economic tension in Palestine has facilitated the spread of illicit drug use among Palestinians. In the context of house demolitions, arrests, restriction of movement and encroachment on land, illicit drugs have been used in Palestine as a ‘coping mechanism’. It provides youth with an opportunity to “escape problems”, obtain a “feeling of relaxation”, to “not think”, and to “fall asleep” [19]. Research on the use of tramadol in the Gaza Strip demonstrates that recurrent attacks and siege related to the Israeli occupation, as well as high unemployment among university graduates are factors associated with the widespread use of tramadol [20]. Similarly, violence and traumatic events experienced by children in the Gaza Strip related to the Israeli occupation is linked to increased illicit drug use among children [21]. In addition to political, economic, and social factors, regulatory and enforcement issues also contribute to the increase of illicit drug use in Palestine, which includes the absence of unified Palestinian authority and police system, internal political turmoil, weak legal enforcement, and limited border control to combat trafficking [22].

Many HRDUs were found to be using drugs, such as Tramadol and sedatives, that should be prescribed by a physician. Based on the UNODC drug report of 2021, there has been a rise in the non-medical use of pharmaceutical drugs between 1995 and 2019 [1]. We found that almost all HRDUs in the Gaza Strip (99%) had used Tramadol (verified through urinalysis) and Lyrica (54%) in the previous month. Although Tramadol and Lyrica are not considered to have the same addiction risk as other narcotics, they are still addictive when used frequently and in high doses [22]. Lyrica was only used in the Gaza Strip. As Lyrica was not yet among the the list of controlled drugs at the time of this study, it was being used instead of Tramadol. Tramadol was classified as a controlled drug, its use was crminalized, and offenders were imprisoned and had to pay to large penalties for each arrest related to Tramadol use .

The majority of HRDUs in the West Bank (south [88%], north [83%], middle region [65%]) reported using synthetic marijuana. Although synthetic marijuana use could not be verified by urinalysis, its use has been increasing rapidly, especially among teenagers and young adults based on survey data and drug seizures since 2013 [6]. According to a study of drug arrests between 2010 and 2014, the most commonly seized and used substances were cannabis, hashish (74.3%) and marijuana (15.2%), followed by synthetic marijuana (26.6%). Although synthetic marijuana has been mistakenly linked to cannabis, it is often easier to obtain, less expensive, and far more dangerous, causing more severe adverse health effects and dependence than cannabis [6]. In the West Bank, synthetic marijuana is mixed with tobacco and with other more toxic compounds including insecticides, rodenticides, and acetone to make it a more potent substance for a lower price [22].

Based on urinalysis, between 12.4% and 21.5% of HRDUs had methamphetamines in their system. The use of amphetamine-type stimulants (ATS), mainly amphetamines, methamphetamines and amphetamine analogues of the MDA-type, including MDMA or Ecstasy, is a growing global problem [23].

A comparison of reported drug use in the previous month with drugs detected in urine, other than marijuana and synthetic marijuana, showed differences in consistency by location. Descrepancies between reported use and the results of the urinalysis may be explained by ignorance of the type of drug used, and the interval of screening as many of the listed substances will not appear in urine after 2–4 days.

The combination of drugs found in this study can produce additive adverse effects on the respiratory and cardiovascular systems. Benzodiazepines and opioids, including heroin, morphine, methadone, codeine and Tramadol, all have central nervous system (CNS) depressant effects. Alcohol is another CNS depressant commonly used in combination with these drugs. Palestine is predominantly a Muslim country, and within Islam alcohol is forbidden (Haram) according to the Qu’ran. Yet, Palestinian law allows for its production and sale.

Amphetamines, cocaine and tricyclic anti-depressants are also associated with risks to the cardiovascular system, including hypertension, life-threatening arrhythmias, and circulatory collapse. Furthermore, the combination of cocaine with alcohol results in increased cardiotoxicity [24]. Studies found that the reason for combining drugs can be intentional for additive recreational effects or can be totally random and unintentional [25, 26]. Regardless of the reason behind it, polydrug use found among the study population is alarming and carries a high risk of additive side effects that could be fatal and can complicate medical interventions in case of toxicity as multiple agents and antidotes could be needed.

Our research found that older HRDUs from the West Bank and the Gaza Strip who were imprisoned were more likely to seek assistance for illicit drug use, which is in line with findings from a previous study conducted among Latinos [8]. This could be explained by use of the rehabilitation services at prison, which are mostly medical, or could also be explained by individuals seeking rehabilitation services to avoid going to prison again. In the West Bank and the Gaza Strip, suffering from the consequences of drug use, memory loss and withdrawal symptoms, respectively, was also associated with seeking rehabilitation services, which is in line with previous research [8], which is comparable to what we observe in the West Bank where individuals who have been imprisoned are more likely to seek such programs.

This study has several strengths. First, it is the first study in Palestine to estimate the extent of high-risk drug use in Palestine among males aged 15 and above, including the extent of polydrug use and the prevalence of ever injecting drugs. Our findings provide evidence to inform prevention and treatment services. Second, our data provide more granular insights into the extent of HRDU by sampling three distinct areas in the West Bank (North, middle, and south), as well as in Gaza. Third, this is the first study to examine factors associated with seeking rehabilitation services among HRDUs to inform policy. Fourth, drug use was verified based on urine analysis, rather than self-report.

However, a major limitation in this study is that females were not included. Females who use illicit drugs are known to be at higher risk than males due to the fact that females have more limited rehabilitation treatment options. Cultural expectations and constraints pose significant barriers to females taking part in any rehabilitation program. In addition, there are inadequate rehabilitation treatment services tailored to the special needs of females who use drugs and who are likely to be more vulnerable to sexual harassment and abuse. Although males who use illicit drugs certainly know females who use illicit drugs, female illicit drug users are normally more hidden and stigmatized than male illicit drug users in Palestine, as in other countries. Nevertheless, it is essential that female illicit drug users are at least qualitatively studied and that appropriate rehabilitation treatment services are available to them. Another potential bias may have been reporting bias due to culture and religion and heightened stigma towards drug and alcohol use. However, we took several precautions to ensure participants felt comfortable, including involving illicit drug users in the planning of the survey, collecting data anonymously, hiring former illicit drug users to screen participants, hiring recruiting trained data collectors, and providing a safe and accepting survey environment. Finally, as participants were not randomly selected, unknown confounders were not adjusted for in the study design. Although sampling begins with non-randomly selected seeds, which introduce a large amount of bias, a goal in RDS is to attain a sufficient number of waves whereby the sample transitions to convergence. Convergence is an indication that the final estimates are no longer impacted by the bias of the seeds and that the final estimates represent the network of the population sampled [15]. In our sample, we reached up to 14 waves and attained convergence on all key variables reported in this paper.

## Conclusion

Our study calculated that there are roughly 26,500 male HRDUs in Palestine comprising 1.8% of the male population aged 15 and above. A high percentage of them are polydrug users and a low percentage of them are injecting drugs. High percentages of HRDUs started using drugs when they were below the ages of 18 years, and most are unaware of existing rehabilitation services. Based on the study findings, a number of policy recommendations regarding illicit drug use in Palestine are evident. These include, educating and providing early interventions to young people who are at higher risk for drug use, developing flexible treatment modalities that involve internationally accepted detoxification and diagnosis, and treatment of co-morbidities such as mental health and drug use related disorders [8], providing appropriate treatment to females, creating plans to ensure the continuity drug use services during emergencies, including during epidemics (i.e., COVID), attacks and sieges, and developing a monitoring system to regularly collect information on drug use and its health consequences to strengthen the evidence base and raise public awareness about the prevention and treatment of drug use. Finally, this study should be repeated over time to assess trends and measure improvements and failures.

## Data Availability

All data generated or analyzed during this study are included in this published article.

## Footnotes

1 Benzodiazepines.

2 Crystal is a street name for methamphetamine.

3 4 Phencyclidine is a dissociative sedative-anesthetic drug with hallucinogenic properties.

## List of abbreviations

HRDU: High risk drug use
RDS: respondent driven sampling
SSPSE: successive sampling population size estimation
UNODC: United Nations Office on Drugs and Crime
